# Pig Feeding

**Published:** 1890-06

**Authors:** 


### PIG FEEDING.

THREE breeds of pigs—Berkshires, Chester Whites and Yorkshires, of two each—were used in a feeding trial. The Chester whites gained about one-fifth faster than the others, required one-seventh less food to produce a pound of increase in live weight than did the others, and produced their growth at a cost in food consumed of three cents per pound, while the other breeds ate three and one-half cents worth of feed for each pound of growth.

The six pigs made an average gain per pig of 1.07 pounds per day, and consumed on an average 2.79 pounds of dry matter to each pound of gain in live weight.

Pigs require more food to produce a pound of growth the older they grow ; hence the longer they are kept the more it costs for each pound of growth and the less profitable they become.

The value of the food consumed for each pound of increase in dressed weight was 4.06 cents, and the fertilizing value of this food 2.54 cents, leaving the net cost of a pound of dressed pork to be 1.52 cents. The meat sold for 5.25 cents a pound, or a net gain of 3.72 cents per pound.

While the tables in this bulletin are clear and interesting, frequently the headings of columns are turned on the side where entirely unnecessary, much to the reader's inconvenience.—*Vermont Agricultural Experiment Station. Bull. No. 18, p. 20.*

